# A Case Report on Metformin-Associated Lactic Acidosis and Transient Blindness

**DOI:** 10.7759/cureus.9325

**Published:** 2020-07-21

**Authors:** Pooja Kalantri, Abhishek Sahu, Aarthi Kalantri

**Affiliations:** 1 Internal Medicine, Saint Vincent Hospital, Worcester, USA; 2 Medicine and Surgery, Osmania Medical College, Hyderabad, IND; 3 Medicine and Surgery, MediCiti Institute of Medical Sciences, Hyderabad, IND

**Keywords:** metformin associated lactic acidosis, blindness, ckd, type 2 diabetes

## Abstract

Metformin is the first-line treatment for any patient with type 2 diabetes. Metformin-associated lactic acidosis and transient blindness have only been reported in some case series and case reports. It is rare and presents especially in patients with underlying chronic kidney disease (CKD) Stage III and above and on high doses of metformin or with a normal dose of metformin and an associated renal injury. We present here a rare and interesting case of something similar. A 77-year-old woman with a past medical history of type 2 diabetes on metformin, obesity status post gastric bypass, CKD Stage III, presented with complaints of nausea, vomiting, confusion, abdominal pain, diarrhea, decreased urine output, sudden visual loss, and a hypoglycemic episode at home. She was hemodynamically stable. Lab work was suggestive of leukocytosis, hyperkalemia, severe high anion gap metabolic and lactic acidosis, acute-on-chronic kidney injury. Findings on the computed tomography (CT) brain, chest radiograph, and CT abdomen and pelvis could not explain the current scenario. She received Ringer’s lactate, a bicarbonate push, and an infusion. Acidosis continued to worsen, she became hypotensive requiring pressor support, and she was immediately taken for hemodialysis. All her symptoms, including vision loss, had improved with a single session of hemodialysis, even before the acidosis had corrected. Work-up for other causes of renal dysfunction came back negative. Metformin was discontinued. She was placed on insulin for her diabetes control.

## Introduction

Metformin-associated lactic acidosis (MALA) and transient blindness are rare, life-threatening phenomena, resulting in hemodynamic instability and severe metabolic abnormalities, usually requiring renal replacement therapy (RRT) [1]. As it has a large volume of distribution, the initiation and duration of dialysis depend on clinical and laboratory targets [2] like the normalization of pH and lactate.

There are studies suggesting that the predialysis level of serum lactate is an important marker of mortality in MALA patients requiring RRT with a linear dose-response relationship [3]. The serum lactate level was significantly higher in non-survivors (median 22.5 mmol/L) than in survivors (17.0 mmol/L, p-value <0.01) and so were the median blood metformin concentrations (58.5 vs. 43.9 mg/L, p-value = 0.05). The survival advantage was not significantly different between the modalities of RRT [3].

So far, the notion was that the removal of metformin by dialysis was uncertain, and it helped correct the associated electrolyte abnormalities [4]. Hence, early recognition and treatment of this complication were very important. But there have been at least three publications suggesting otherwise. Most published reports present incomplete data: metformin clearance with high-efficiency hemodialysis (HD) surpasses 120 ml/min and has a metformin half-life that is approximately four hours. In comparison, metformin clearance with continuous renal replacement therapy is usually about one-fourth of that obtained with HD, and the half-life is at least three times as long. However, because of metformin’s large volume of distribution, the significance of these data is debatable without quantifying metformin removal. Only three publications have presented data indicating metformin removal by extracorporeal treatment. Lalau and Race reported the removal of 1105 mg, 694 mg, and 688 mg by HD in three patients [5]. Barrueto et al. reported 3.5 g removal by continuous venovenous hemodialysis in 10.5 hours [6], and Ayoub et al. reported the removal of 1039 mg in 5.5 hours during hemodialysis session one and another 463 mg in 6.2 hours during hemodialysis session two [4].

## Case presentation

A 77-year-old female, with a past medical history of hypertension, obesity status post gastric bypass, chronic kidney disease (CKD) stage III, anemia of CKD, hyperlipidemia, and type 2 diabetes for 48 years, on 1250 mg twice daily metformin, presented to the hospital after she was found acutely confused at home with sudden-onset vision loss. She also complained of nausea, vomiting, poor appetite, abdominal pain, diarrhea, and decreased urine output for a few days. The blood sugar level was in the 34 mg/dL before arrival, which was treated en route.

In the emergency department, she was hemodynamically stable. She was alert and oriented to time, place, and person, with no focal neurological deficits except for the vision loss. Laboratory analysis was concerning for leukocytosis of 21,000 cells/microlitre, potassium 5.9 mEq/L, bicarbonate 3 mEq/L, blood urea nitrogen (BUN) 51 mg/dL, creatinine 4.39 mg/dL (with a baseline of 1.4 mg/dL), estimated-glomerular filtration rate (e-GFR) <30 mL/min, and lactate 23.8 mmol/L. The rest of the complete blood counts, basic metabolic panel, liver function tests, creatine kinase, troponin, urine analysis, urine toxicology, and urine alcohol levels were unremarkable. Electrocardiography (EKG) showed no changes in hyperkalemia. CT abdomen and pelvis showed a 4 mm non-obstructing calculus in the right kidney with no hydronephrosis. CT brain and chest X-ray were normal. She received a liter of Ringer’s lactate following which she was started on a bicarbonate push and drip. With worsening acidosis, due to unclear reasons at the time, with an arterial blood gas (ABG) showing pH less than 7, and bicarbonate unreadable, she was transferred to the intensive care unit (ICU) for urgent hemodialysis.

In the ICU, while undergoing HD, she was transiently hypotensive requiring two pressor supports for around eight hours. She also complained of respiratory distress. She was placed on bilevel positive airway pressure (BiPAP) intermittently, for the same. Repeat ABG after completion of hemodialysis showed a pH of 7.45, pCO2 24.8 mm/Hg, pO2 124.1 mm/Hg while the HCO3 was still 18.7 mEq/L. The patient's mental status improved and vision came back to normal after a four-hour session of hemodialysis. She was also back to room air from the transient BiPAP support. Her toxic metabolic encephalopathy was thought to be secondary to metformin toxicity in the setting of chronic kidney disease.

On the medical floor, she was slightly volume overloaded. BUN and creatinine continued to rise to reach a peak of 59 and 6.16, respectively. She also had 800 mg of proteinuria, likely related to her underlying diabetes. Serum and urine immunofixation were normal. Antinuclear antibody (ANA), cytoplasmic-antineutrophil cytoplasmic antibodies (c-ANCA), perinuclear-antineutrophil cytoplasmic antibodies (p-ANCA), anti-glomerular basement membrane (anti-GBM) antibody, and complements were all normal. While a plan was being made regarding a renal biopsy, her creatinine started to plateau and she had a good urine output. At discharge, she had a creatinine of five. Her metformin was discontinued and she was started on insulin for her diabetes. Ultimately, in a few months, she recovered her renal function to a serum creatinine of 1.8-1.9 mg/dL, a little higher than her baseline.

## Discussion

Metformin is the first-line treatment for type 2 diabetes [3]. Many patients with diabetes have underlying CKD. Based on the current recommendations, the use of this medication is acceptable, with an eGFR above 45 mL/min. Between the eGFR of 30 and 45, some recommend not initiating the medication while others prefer starting it at half the usual dose with monitoring of renal function every three months. Below the eGFR of 30, its use is contraindicated [7].

Blood concentrations of metformin ranging between 0.5 and 2.0 mg/L are within the therapeutic range, whereas those higher than 4.0 mg/L are generally considered to be toxic. The lethal range of metformin concentrations is >50 mg/L [8].

Metformin mostly causes gastrointestinal side effects, including diarrhea, flatulence, nausea, vomiting, and infections [8]. Other side effects include chest discomfort, flushing, palpitations, diaphoresis, cyanocobalamin deficiency, hypoglycemia, headache, and rhinitis. Lactic acidosis and blindness are rare, and their incidence is not clear, as they are reported only in some case reports and case series [9]. The most common symptoms of MALA are those related to the gastrointestinal tract (including nausea, vomiting, and diarrhea) followed by an altered mental status, shortness of breath, hypothermia, and hypotension [8].

According to previously published reports, vision loss associated with MALA improved after the correction of metabolic acidosis [10]. Based on animal studies, the suggested mechanism involves the following: retinal cell function may be pH-dependent; retinal horizontal cell response to light is pH-sensitive; and mammalian retinal cell function becomes disrupted at pH <7.09. These effects could extend to humans and may serve as an explanation for acidosis-associated vision loss and optic nerve ischemia [11]. But, in our case, the vision improved even before the acidemia corrected. We suggest that since acute reversible blindness has been described with MALA, but not in patients with hypotension- or sepsis-induced lactic acidosis, this neurologic symptom may be a direct result of abnormal retinal horizontal cell function induced by metformin at a low pH, or the metabolic effects of metformin, rather than due to the acidemia alone.

Diagnosis and therapy may be delayed due to nonspecific symptoms at presentation, with severe anion gap metabolic acidosis and elevated serum creatinine values being the most prominent laboratory findings. MALA is characterized by a blood lactate concentration greater than 5 mmol/L and arterial pH less than 7.35 in association with metformin exposure [9]. Confirmation requires measurement of serum metformin by high-performance liquid chromatography-tandem mass spectrometry, but this technique is available only at specialized institutions and cannot be relied on as a guide to immediate treatment [12]. 

Thus, based on strong clinical suspicion, renal replacement therapy must be started promptly to achieve efficient drug clearance and correct the metabolic acidosis. However, because metformin accumulates in the intracellular compartment with prolonged treatment, a rebound in serum concentrations due to redistribution is expected at the end of dialysis [12].

In our case, the patient was a diabetic for almost 48 years, on metformin. She likely had an underlying prerenal AKI, which predisposed her to metformin toxicity. She had the classic clinical and laboratory abnormalities of the same. Though a blood level was not checked to confirm the diagnosis, her clinical picture and lab abnormalities had all corrected with the correction of her metabolic derangements. She did not require further sessions of hemodialysis. Her metformin was discontinued, and she was instead started on insulin for her diabetes, with other oral agents to be considered in an outpatient setting.

## Conclusions

Metformin should be discontinued in all cases of MALA and vision loss. Other hypoglycemic agents should instead be considered to prevent this life-threatening complication from occurring again. Vision improves after the correction of acidosis but, in our case, the vision improved even before the acidemia corrected. We suggest that since acute reversible blindness has been described with MALA, but not in patients with hypotension- or sepsis-induced lactic acidosis, this neurologic symptom may be a direct result of abnormal retinal horizontal cell function induced by metformin at a low pH, or the metabolic effects of metformin, rather than due to the acidemia alone. This needs to be studied further. Early treatment in the form of renal replacement therapy should be considered in any suspected case, especially with lactate levels greater than 17 mmol/L to prevent mortality and other complications like vision loss. The mainstay of renal replacement therapy is to correct metabolic derangements. Further studies regarding the metformin clearance by hemodialysis are also warranted.
